# Association of Inadequate Caloric Supplementation with 30-Day Mortality in Critically Ill Postoperative Patients with High Modified NUTRIC Score

**DOI:** 10.3390/nu10111589

**Published:** 2018-10-29

**Authors:** Yun Tae Jung, Jung Yun Park, Jiyeon Jeon, Myung Jun Kim, Seung Hwan Lee, Jae Gil Lee

**Affiliations:** 1 Department of Surgery, School of Medicine, Ajou University, Suwon 16499, Korea; paysan@aumc.ac.kr; 2 Department of Surgery, Yonsei University College of Medicine, Seoul 03722, Korea; sinistraza@yuhs.ac (J.Y.P.); mj4jina@yuhs.ac (M.J.K.); seunghwan@yuhs.ac (S.H.L.); 3 Yonsei University College of Nursing, Seoul 03722, Korea; ioio09161@gmail.com

**Keywords:** nutrition, mortality, calorie adequacy, critically ill, abdominal surgery, NUTRIC

## Abstract

Modified NUTRIC (mNUTRIC) score is a useful assessment tool to determine the risk of malnutrition in patients on mechanical ventilation (MV). We identified associations between postoperative calorie adequacy, 30-day mortality, and surgical outcomes in patients with high mNUTRIC scores. Medical records of 272 patients in the intensive care unit who required MV support for >24 h after emergency gastro-intestinal (GI) surgery between January 2007 and December 2017 were reviewed. Calorie adequacy in percentage (Calorie intake in 5 days ÷ Calorie requirement for 5 days × 100) was assessed in patients with high (5–9) and low (0–4) mNUTRIC scores. In the high mNUTRIC score group, patients with inadequate calorie supplementation (calorie adequacy <70%) had higher 30-day mortality than those with adequate supplementation (31.5% vs. 11.1%; *p =* 0.010); this was not observed in patients with low mNUTRIC scores. This result was also confirmed through Kaplan–Meier survival curve (*p* = 0.022). Inadequate calorie supplementation in the high mNUTRIC score group was not associated with Intra-abdominal infection (*p* = 1.000), pulmonary complication (*p* = 0.695), wound complication (*p* = 0.407), postoperative leakage (*p* = 1.000), or infections (*p* = 0.847). Inadequate calorie supplementation after GI surgery was associated with higher 30-day mortality in patients with high mNUTRIC scores. Therefore, adequate calorie supplementation could contribute to improved survival of critically ill postoperative patients with high risk of malnutrition.

## 1. Introduction

Adequate nutritional provision is important for critically ill patients [1,2,3]. Kondrup et al. [4] showed that adequate nutrition support for these patients resulted in improved clinical outcomes. In particular, patients at ‘high risk’ for malnutrition showed significant improvements, while ‘low risk’ patients experienced minimal effects of nutrition support. Therefore, identifying patients who are at high risk of malnutrition is an important role of intensivists and nutrition practitioners.

It has always been challenging for intensivists to evaluate nutritional risk of intensive care unit (ICU) patients. Critically ill patients are in severe catabolic stress and have pro-inflammatory status due to increased release of stress-related hormones and cytokines [5]. Therefore, the severity of their disease, which could affect the severity of catabolism and protein loss, should be considered in their nutritional risk assessment [4]. Many ICU patients are on mechanical ventilation (MV), with no ability to communicate information about their previous nutritional status to clinicians. Although several nutritional risk assessment tools are used in clinical practice [4,6,7,8], these tools consider all ICU patients as having a high risk of malnutrition [4,6,9]. Besides, these tools include a subjective questionnaire which is relatively difficult to obtain from patients under MV.

The NUTrition RIsk in the Critically ill (NUTRIC) score [10] is designed for assessing the nutritional risk of patients in the ICU who are receiving MV. The NUTRIC score is designed on the concept that age, inflammatory status, and starvation status will affect a patient’s nutritional status, micronutrient deficiency, immune dysfunction, and clinical outcome. NUTRIC score consists of 6 variables, which are age, acute physiology and chronic health evaluation (APACHE) II score, sequential organ failure assessment (SOFA) score, number of co-morbidities, days from hospital to ICU admission, and interleukin-6 (IL-6). Patients get 1–3 points for each variables and the highest score adds up to 10. A study by Heyland et al. [10] showed that patients with high nutritional risk, as identified by the NUTRIC score, received the most benefit from adequate nutritional support. However, the IL-6 level is not commonly measured in many institutions. The modified NUTRIC score (mNUTRIC) excludes the use of the IL-6 level and is easier to calculate, and it has been validated to help discriminate which patients will benefit more from energy provision [11].

Several studies were conducted to validate mNUTRIC score [10,11,12] but no study focused on surgical ICU patients only. Patients who undergo emergency abdominal operations are different from those in the medical ICU because of functional impairment of gastro-intestinal (GI) tract conjoined with operative manipulation, edema, or inflammation. These factors can delay initiation of enteral diet. Therefore, our study was designed to evaluate the benefit of adequate caloric supplementation for these patients who had undergone emergency GI surgery and had a high risk of malnutrition, using the mNUTRIC score for risk stratification.

## 2. Materials and Methods

### 2.1. Study Population

This study was conducted retrospectively in a university-affiliated hospital in the Republic of Korea. Electronic medical records of 1118 adult patients who underwent GI surgery for complicated intra-abdominal infection from January 2007 through December 2017 were reviewed. A total of 272 patients who required postoperative MV therapy for more than 24 h were included for analysis. Five patients who died within 72 h after surgery were excluded from analysis.

Patients who underwent GI surgery for complicated intra-abdominal infection were chosen because complicated intra-abdominal infection is one of the most common surgical condition which causes critical illness of affected patients. Most of them are in sepsis and septic shock, and they commonly stay in ICU for a long time and receive MV therapy. Therefore, this population is homogeneous and ideal for evaluation of survival benefit of adequate caloric supplementation.

**Figure 1:**
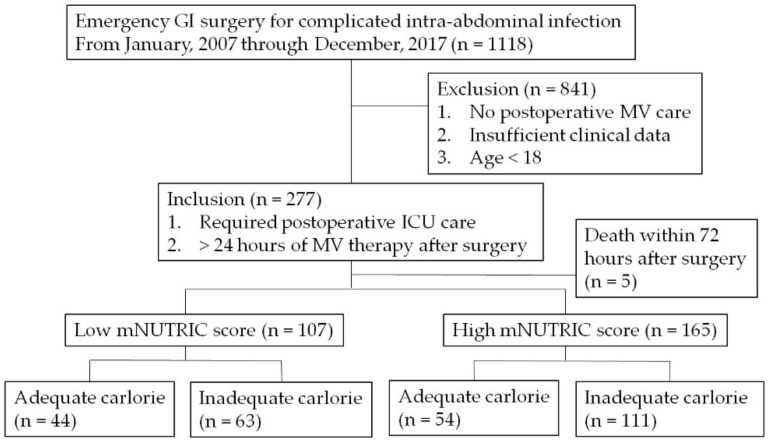
Flow diagram of patients selected for analysis. GI: gastro-intestinal; MV: mechanical ventilation; ICU: intensive care unit; mNUTRIC: modified NUTrition RIsk in the Critically ill.

Patients were considered to be at high risk of malnutrition and to benefit from aggressive nutritional support when their mNUTRIC score was ≥5. Patients were divided into high- and low-risk groups of malnutrition according to their mNUTRIC scores. Patients in the high-risk group were divided into two sub-groups according to the adequacy of their caloric intake: inadequate nutrition group and adequate nutrition group (Figure 1). Patients with low mNUTRIC score were also divided into the two subgroups, and results are demonstrated in a (Supplementary File, Supplementary Tables S1–S4).

### 2.2. Data Collection and Definition

Patients’ baseline characteristics and all components of the SOFA score and APACHE II score were collected at the day of their ICU admission. Calorie requirements are calculated as 25 kcal/kg as referred in the guidelines [13,14]. Calorie intake were calculated for a maximum of 5 days from admission. Days were excluded from the calculation when a patient started oral diet because patients usually start oral diet after transfer to general wards, and their condition are no longer considered as critical. Moreover, when patients start oral diet, it is difficult to quantify their caloric intake. Days were also excluded when a patient died before day five. Calorie adequacy was calculated as a percentage of caloric intake divided by caloric requirement during the days of calculation (Calorie adequacy (%) = Calorie intake in 5 days ÷ Calorie requirement for 5 days × 100). Inadequate calorie supplementation was defined as calorie adequacy less than 70%. Application of continuous renal replacement therapy (CRRT), event of hypotension, and vasopressor use were also reviewed during the data calculation period. The primary clinical outcome we wanted to evaluate in this study was 30-day mortality. Furthermore, MV-free days, ICU-free days, and hospital lengths of stay (HLOS) were analyzed. Postoperative complications, such as intra-abdominal infection (IAI), pulmonary complication, wound complication, postoperative leakage, and postoperative infections were also evaluated.

Enteral nutrition (EN) was started as soon as possible, when patients were hemodynamically stable, not receiving high doses of vasopressors, secure in their bowel anastomosis or repair sites, and not having signs of delayed gastric emptying [15]. EN was provided through either Levin tube or oral route according to their mental and functional status, and independency from MV. A role of early supplemental parenteral nutrition (PN) or early provision of combined EN/PN for immediate postoperative patients are not clearly established yet [16,17,18]. Therefore, it was provided on the preferences of the responsible surgeon for each patient.

This study was approved by the Institutional Review Board of Severance Hospital, Yonsei University Health System (4-2017-0944), and the need for informed consent was waived due to the retrospective study design.

### 2.3. Statistical Analysis

Continuous variables were expressed as mean ± standard deviation and compared using the *t*-test or Mann–Whitney U test, as appropriate. Categorical variables were expressed as frequency (percentage) and compared using chi-square or Fisher’s exact test. Baseline differences of the patients between groups were adjusted using the propensity score-matching method. Age, sex, body mass index (BMI), American Society of Anesthesiologists (ASA, Schaumburg, IL, USA), SOFA score, and application of CRRT were the baseline variables adjusted in this matching process. After matching, the Kaplan–Meier survival curve and log-rank test were used to compare 30-day mortality between the sub-groups to demonstrate the survival benefit of adequate caloric supplementation in patients with a high risk of malnutrition. SPSS^®^ Statistics 23.0 (IBM Corp., Armonk, NY, USA) was used for statistical analysis. R-software (version 3.4.3, R Foundation for Statistical Computing, Vienna, Austria) and MatchIt package were used for propensity score matching.

## 3. Results

### 3.1. Baseline Characteristics

Of 272 patients, 107 patients had low mNUTRIC scores (<5), and 165 patients had high mNUTRIC scores (≥5). The study results are focused on patients with high mNUTRIC scores. Fifty-four patients received adequate nutrition, and 111 patients received inadequate nutrition among patients with high mNUTRIC scores. Patients who received inadequate calorie supplementation had a higher BMI (23.17 ± 4.15 vs. 21.03 ± 3.11; *p* < 0.001), ASA score (*p* = 0.049), and SOFA score (7.86 ± 3.07 vs. 6.56 ± 3.04; *p* = 0.011). These differences were adjusted after the matching process (Table 1).

### 3.2. Perioperative Parameters

Event of hypotension (86.5% vs. 77.8%; *p* = 0.156), vasopressor use (71.2% vs. 57.4%; *p* = 0.078), and application of CRRT (36.9% vs. 22.2%; *p* = 0.058) seemed to occur more often in the group with inadequate calorie supplementation, but the differences were not statistically significant, and the findings did not change after the matching process (Table 2).

### 3.3. Calorie Requirement and Adequacy

Patients in the inadequate nutrition group had higher calorie requirements than patients in the adequate nutrition group (1513.45 ± 321.64 vs. 1327.87 ± 223.92; *p* < 0.001). The difference between groups was minimized after the matching process (1366.34 ± 282.91 vs. 1327.87 ± 223.92; *p* = 0.435). Average calorie adequacy in the inadequate nutrition group and the adequate nutrition group were 44.1% and 88.1%, respectively (46.5% and 88.1%, respectively, after matching). Approximately one in five patients with a high mNUTRIC score started EN within 5 days of admission to the ICU (23.4% vs. 20.4%; *p* = 0.659); the findings were similar after the matching process (24.1% vs. 20.4%; *p* = 0.643). The adequate nutrition group received more PN supplementation within 5 days of admission than the inadequate nutrition group (87.0% vs. 48.6%; *p* < 0.001); the difference remained in the matched population (87.0% vs. 48.1%; *p* < 0.001) (Table 3).

### 3.4. Clinical Outcomes

**Figure 2:**
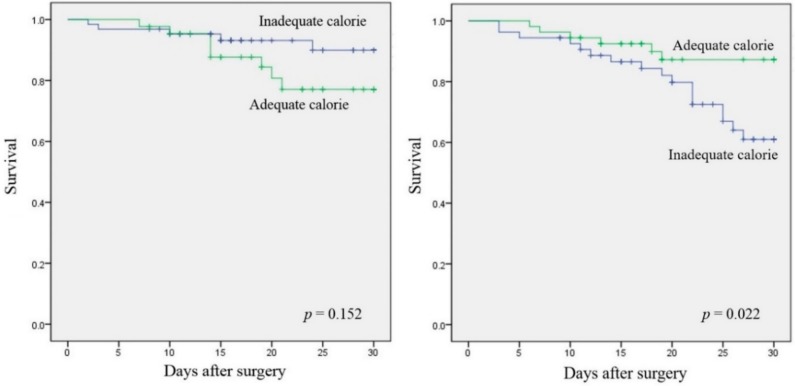
Kaplan–Meier survival curves for adequate and inadequate calorie supplementation in patients with low mNUTRIC score (**left**) and high mNUTRIC score (**right**); propensity score matching was performed for high mNUTRIC score group. The curves on the left demonstrate that there is no survival benefit after adequate calorie supplementation in patients with low mNUTRIC (log-rank test, *p* = 0.152), but the curves on the right demonstrate that there is a significant survival benefit in the adequate nutrition group (log-rank test, *p* = 0.022).

In the low mNUTRIC score group, 30-day mortality rate was not higher in the inadequate nutrition group than in the adequate nutrition group (Supplementary Table S4). In contrast, the 30-day mortality rate was higher in the inadequate nutrition group than in the adequate nutrition group in the high mNUTRIC score group (34.2% vs. 11.1%; *p* = 0.002); this was also shown in the matched population (31.5% vs. 11.1%; *p* = 0.010). The Kaplan–Meier survival curve also demonstrated a survival benefit in the adequate nutrition group in the high mNUTRIC score group, even after matching (log-rank test, *p* = 0.022); this was not shown in the low mNUTRIC score group (log-rank test, *p* = 0.152) (Figure 2). Nevertheless, inadequate nutrition was not associated with other outcomes, such as MV-free days, ICU-free days, and HLOS in both low and high mNUTRIC score group. Furthermore, postoperative complications, such as IAI, pulmonary complication, wound complication, postoperative leak, and infection seemed to have no association with nutritional adequacy (Table 4).

## 4. Discussion

Our data demonstrated that provision of adequate nutrition for critically ill patients with high mNUTRIC scores is associated with better survival. Furthermore, patients with a high risk of malnutrition appeared to receive the greatest benefit from adequate nutritional support. This trend has been shown by several previous studies [4,10,11]. We focused on the data from patients with high mNUTRIC scores because patients with low mNUTRIC scores showed no survival benefit from adequate nutritional support. The results for this patient group are demonstrated in a (Supplementary File, Supplementary Tables S1–S4).

Sequential organ failure assessment (SOFA) score and American Society of Anesthesiologists (ASA) score have been shown to be good predictors of patient mortality [19,20,21]. Our results showed differences in these scores between adequate and inadequate nutrition groups. Differences in baseline characteristics that could influence patient outcomes need to be matched; we used the propensity score-matching method. Even after the matching process, a clear association was found between 30-day mortality and receiving inadequate calorie supplementation in the high mNUTRIC score group. However, adequate calorie supplementation did not influence other surgical outcomes, such as IAI, pulmonary or wound complication, postoperative leakage, and infection. There are different results from studies on the relationship between infection rates and calorie adequacy in critically ill patients [22,23,24]. However, infection in surgical patients may be influenced not only by adequacy of calorie supplementation, but also by many other factors, such as degree of contamination in the operation site, duration of endotracheal intubation, and combination and duration of antibiotic therapy. Wound complication and postoperative leakage might be more associated with techniques used during surgery, extent and severity of infection source, or wound management techniques, rather than the method used to supply nutrition to patients in the ICU.

Postoperative patients have a lower tolerance for oral diet than other critically ill patients, and without PN, they are often malnourished [25,26,27]. In our study, nearly half of the patients did not reach 80% of the caloric requirements with EN only, and only 20% of the patients started EN within 5 days. There are still controversies between hypocaloric versus normocaloric nutrition for critically ill patients [1,10,22,24]. However, providing adequate calories for surgical patients with a high risk of malnutrition who underwent emergency GI surgery was associated with better clinical outcomes in our study. In patients with high mNUTRIC scores and GI intolerance after surgery, PN can be an option for adequate energy supplementation. Because there is not enough evidence on the role of parenteral supplementation to achieve near caloric goal in surgical patients who are at high risk of malnutrition, further studies should be performed to understand the benefit of early parenteral supplementation in this population.

This study has several limitations. First, our data are based on a single-center retrospective study in a surgical ICU, and thus our findings may not be generalizable to other populations. Secondly, indirect calorimetry is ideal for calculating the energy requirement of individuals, but it was not available during the study period. As an alternative, we used a formula to calculate required energy using the actual bodyweight of each patient. Our study also did not consider protein adequacy, which can still affect the clinical outcomes. There were no adequate data in the electronic medical records that provide information on protein provision for the study population due to the retrospective nature of this study. Further studies using indirect calorimetry for calculation of actual and real-time energy requirements may be helpful to confirm our results. Additionally, future studies are needed to better understand the beneficial effects of micronutrients and protein provisions for critically ill surgical patients with a high risk of malnutrition who would most benefit from an adequate energy provision.

## 5. Conclusions

Inadequate calorie supplementation after emergency GI surgeries was associated with higher 30-day mortality in patients with high mNUTRIC scores. Adequate calorie supplementation for critically ill postoperative patients with high nutritional risk could contribute to improved patient survival.

## Figures and Tables

**Table 1:** Baseline characteristics of patients with high mNUTRIC scores.

Variable	Total PopulationHigh Risk (mNUTRIC ≥ 5) (*n* = 165)			Matched PopulationHigh Risk (mNUTRIC ≥ 5) (*n* = 108)		
	Inadequate Nutrition (*n* = 111)	Adequate Nutrition (*n* = 54)	*p* Value	Inadequate Nutrition (*n* = 54)	Adequate Nutrition (*n* = 54)	*p* Value
Age, *n*	71.02 ± 10.97	69.52 ± 11.48	0.418	69.96 ± 11.52	69.52 ± 11.48	0.841
Sex, M/F, *n* (%)	66 (59.5)/45 (40.5)	23 (42.6)/31 (57.4)	0.041	23 (42.6)/31 (57.4)	23 (42.6)/31 (57.4)	1.000
BMI, kg/m^2^	23.17 ± 4.15	21.03 ± 3.11	<0.001	21.02 ± 4.47	21.03 ± 3.11	0.603
Weight, kg	60.65 ± 12.80	53.11 ± 8.96	<0.001	54.65 ± 11.32	53.11 ± 8.96	0.435
Height, *m*	1.62 ± 0.09	1.59 ± 0.08	0.081	1.60 ± 0.10	1.59 ± 0.08	0.679
ASA, *n* (%)			0.049			0.627
1	9 (8.1)	12 (22.2)		7 (13.0)	12 (22.2)	
2	9 (8.1)	8 (14.8)		7 (13.0)	8 (14.8)	
3	50 (45.0)	18 (33.3)		25 (46.3)	18 (33.3)	
4	39 (35.1)	14 (25.9)		13 (24.1)	14 (25.9)	
5	4 (3.6)	2 (3.7)		2 (3.7)	2 (3.7)	
APACHE II, *n*	30.48 ± 6.85	29.43 ± 5.78	0.332	29.41 ± 5.81	29.43 ± 5.78	0.987
SOFA, *n*	7.86 ± 3.07	6.56 ± 3.04	0.011	6.89 ± 3.17	6.56 ± 3.04	0.578
NUTRIC, *n*	6.03 ± 1.07	5.94 ± 0.98	0.632	5.80 ± 0.88	5.94 ± 0.98	0.409
HTN, *n* (%)	67 (60.4)	28 (51.9)	0.299	29 (53.7)	28 (51.9)	0.847
DM, *n* (%)	35 (31.5)	13 (24.1)	0.322	13 (24.1)	13 (24.1)	1.000
CRF, *n* (%)	13 (11.7)	7 (13.0)	0.817	5 (9.3)	7 (13.0)	0.540
Cancer, *n* (%)	60 (54.1)	26 (48.1)	0.476	37 (68.5)	26 (48.1)	0.032

mNUTRIC: modified NUTrition Risk in Critically ill; M/F: male/female; BMI: body mass index; APACHE: acute physiology and chronic health evaluation; SOFA: sequential organ failure assessment; ASA: American Society of Anesthesiologists; NUTRIC: NUTrition RIsk in Critically ill; HTN: Hypertension; DM: Diabetes mellitus; CRF: Chronic renal failure.

**Table 2:** Perioperative parameters of patients with high mNUTRIC scores.

Variable	Total PopulationHigh Risk (mNUTRIC ≥ 5) (*n* = 165)			Matched PopulationHigh Risk (mNUTRIC ≥ 5) (*n* = 108)		
	Inadequate Nutrition (*n* = 111)	Adequate Nutrition (*n* = 54)	*p* Value	Inadequate Nutrition (*n* = 54)	Adequate Nutrition (*n* = 54)	*p* Value
SBP < 100, *n* (%)	96 (86.5)	42 (77.8)	0.156	45 (83.3)	42 (77.8)	0.466
Vasopressors use, *n* (%)	79 (71.2)	31 (57.4)	0.078	35 (64.8)	31 (57.4)	0.430
CRRT, *n* (%)	41 (36.9)	12 (22.2)	0.058	12 (22.2)	12 (22.2)	1.000
Diagnosis, *n* (%)			0.894			0.940
Perforation	80 (72.1)	40 (74.1)		41 (75.9)	40 (74.1)	
Strangulation	18 (16.2)	9 (16.7)		9 (16.7)	9 (16.7)	
Ischemia	13 (11.7)	5 (9.3)		4 (7.4)	5 (9.3)	
Primary infection source, *n* (%)			0.769			0.818
Stomach	19 (17.1)	9 (16.7)		9 (16.7)	9 (16.7)	
Small bowel	41 (36.9)	23 (42.6)		20 (37.0)	23 (42.6)	
Colorectal	51 (45.9)	22 (40.7)		25 (46.3)	22 (40.7)	
Laparoscopy/Open, *n* (%)	8 (7.2)/103 (92.8)	4 (7.4)/50 (92.6)	0.963	4 (7.4)/50 (92.6)	4 (7.4)/50 (92.6)	1.000

mNUTRIC: modified NUTrition Risk in Critically ill; SBP: systolic blood pressure; CRRT: Continuous renal replacement therapy.

**Table 3:** Calorie requirement and adequacy of patients with high mNUTRIC scores.

Variable	Total PopulationHigh Risk (mNUTRIC ≥ 5) (*n* = 165)			Matched PopulationHigh Risk (mNUTRIC ≥ 5) (*n* = 108)		
	Inadequate Nutrition (*n* = 111)	Adequate Nutrition (*n* = 54)	*p* Value	Inadequate Nutrition (*n* = 54)	Adequate Nutrition (*n* = 54)	*p* Value
Required calorie, kcal	1513.45 ± 321.64	1327.87 ± 223.92	<0.001	1366.34 ± 282.91	1327.87 ± 223.92	0.435
Calorie adequacy, %	44.07 ± 14.82	88.12 ± 17.37	<0.001	46.50 ± 13.39	88.12 ± 17.37	<0.001
EN within 5 days, *n* (%)	26 (23.4)	11 (20.4)	0.659	13 (24.1)	11 (20.4)	0.643
PN supplement within 5 days, *n* (%)	54 (48.6)	47 (87.0)	<0.001	26 (48.1)	47 (87.0)	<0.001

mNUTRIC: modified NUTrition Risk in Critically ill; EN: enteral nutrition; PN: parenteral nutrition.

**Table 4:** Clinical outcomes of patients with high mNUTRIC scores.

Variables	Total PopulationHigh Risk (mNUTRIC ≥ 5) (*n* = 165)			Matched PopulationHigh Risk (mNUTRIC ≥ 5) (*n* = 108)		
	Inadequate Nutrition (*n* = 111)	Adequate Nutrition (*n* = 54)	*p* Value	Inadequate Nutrition (*n* = 54)	Adequate Nutrition (*n* = 54)	*p* Value
In-hospital mortality, *n* (%)	48 (43.2)	13 (24.1)	0.017	21 (38.9)	13 (24.1)	0.097
30-day mortality, *n* (%)	38 (34.2)	6 (11.1)	0.002	17 (31.5)	6 (11.1)	0.010
MV-free day, median (Q1, Q3), d	27 (12.0, 29.0)	26 (18.0, 29.0)	0.993	25 (17.0, 28.0)	28 (21.0, 29.0)	0.131
ICU-free day, (Q1, Q3), d	22 (1.5, 26.0)	22.5 (9.0, 26.0)	0.747	20.5(6.0, 26.0)	24 (7.0, 26.0)	0.535
HLOS, median (Q1, Q3), d	22 (14.0, 36.0)	24 (16.0, 50.0)	0.227	23.5 (15.0, 39.0)	24 (16.0, 50.0)	0.645
IAI, *n* (%)	31 (27.9)	13 (24.1)	0.599	13 (24.1)	13 (24.1)	1.000
Pulmonary complication, *n* (%)	60 (54.1)	31 (57.4)	0.684	31 (57.4)	33 (61.1)	0.695
Wound complication, *n* (%)	36 (32.4)	19 (35.2)	0.725	15 (27.8)	19 (35.2)	0.407
Postoperative leak, *n* (%)	19 (17.1)	6 (11.1)	0.313	6 (11.1)	6 (11.1)	1.000
Infection, *n* (%)	64 (57.7)	28 (51.9)	0.481	27 (50.0)	28 (51.9)	0.847

mNUTRIC: modified NUTrition Risk in Critically ill; MV: mechanical ventilation; ICU: intensive care unit; HLOS: hospital length of stay; IAI: intra-abdominal infection.

## Supplementary Materials

The following are available online at http://www.mdpi.com/2072-6643/10/11/1589/s1, Table S1: Baseline characteristics of patients with low mNUTRIC scores, Table S2: Perioperative parameters of patients with low mNUTRIC scores, Table S3: Calorie requirement and adequacy in patients with low mNUTRIC scores, Table S4: Clinical outcomes in patients with low mNUTRIC scores.
