# Supplementary material for: Association of Inadequate Caloric Supplementation with 30-Day Mortality in Critically Ill Postoperative Patients with High Modified NUTRIC Score
Source: Nutrients. 2018 Oct 29;10(11):1589. doi: 10.3390/nu10111589 (PMC6266175; doi:10.3390/nu10111589)
Supplement: Supplementary file 1 [file nutrients-10-01589-s001.pdf]

**Table S1.** Baseline characteristics of patients with low mNUTRIC scores.

| Variable | Low Risk (mNUTRIC < 5) (n = 107) | | |
|---|---|---|---|
| | **Inadequate nutrition (n = 63)** | **Adequate nutrition (n = 44)** | ***p* value** |
| Age, n | 56.98 ± 16.69 | 57.91 ± 13.98 | 0.764 |
| Sex, M/F, n(%) | 45 (71.4)/18 (28.6) | 27 (61.4)/17 (38.6) | 0.275 |
| BMI, kg/m$^2$ | 23.10 ± 3.93 | 20.36 ± 3.02 | <0.001 |
| Weight, kg | 62.83 ± 13.23 | 54.88 ± 10.15 | 0.001 |
| Height, m | 1.65 ± 0.10 | 1.64 ± 0.08 | 0.676 |
| ASA, n(%) | | | 0.199 |
| 1 | 12 (19.0) | 13 (29.5) | |
| 2 | 10 (15.9) | 10 (22.7) | |
| 3 | 29 (46.0) | 11 (25.0) | |
| 4 | 11 (17.5) | 10 (22.7) | |
| 5 | 1 (1.6) | 0 (0.0) | |
| APACHE II, n | 20.11 ± 7.07 | 21.98 ± 6.82 | 0.176 |
| SOFA, n | 4.79 ± 2.63 | 4.41 ± 2.51 | 0.385 |
| NUTRIC, n | 3.02 ± 1.07 | 2.95 ± 1.03 | 0.768 |
| HTN, n(%) | 23 (36.5) | 6 (13.6) | 0.009 |
| DM, n(%) | 6 (9.5) | 1 (2.3) | 0.236 |
| CRF, n(%) | 3 (4.8) | 0 (0.0) | 0.267 |
| Cancer, n(%) | 24 (38.1) | 24 (54.5) | 0.092 |

mNUTRIC: modified NUTrition Risk in Critically ill; M/F: male/female; BMI: body mass index; APACHE: acute physiology and chronic health evaluation; SOFA: sequential organ failure assessment; ASA: American Society of Anesthesiologists; NUTRIC: NUTrition Risk in Critically ill; HTN: Hypertension; DM: diabetes mellitus; CRF: chronic renal failure.

**Table S2.** Perioperative parameters of patients with low mNUTRIC scores.

| Variable | Low Risk (mNUTRIC < 5) (n = 107) | | |
|---|---|---|---|
| | **Inadequate nutrition (n = 63)** | **Adequate nutrition (n = 44)** | ***p* value** |
| SBP<100, n(%) | 29 (46.0) | 25 (56.8) | 0.272 |
| Vasopressors use, n(%) | 16 (25.4) | 21 (47.7) | 0.017 |
| CRRT, n(%) | 6 (9.5) | 6 (13.6) | 0.616 |
| Diagnosis, n(%) | | | 0.719 |
| Perforation | 48 (76.2) | 36 (81.8) | |
| Strangulation | 11 (17.5) | 5 (11.4) | |
| Ischemia | 4 (6.3) | 3 (6.8) | |
| Primary infection source, n(%) | | | 0.167 |
| Stomach | 7 (11.1) | 11 (25.0) | |
| Small bowel | 25 (39.7) | 15 (34.1) | |
| Colorectal | 31 (49.2) | 18 (40.9) | |
| Laparoscopy /Open, n(%) | 8 (12.7)/55 (87.3) | 3 (6.8)/41 (93.2) | 0.519 |

mNUTRIC: modified NUTrition Risk in Critically ill; SBP: systolic blood pressure; CRRT: Continuous renal replacement therapy.

**Table S3.** Calorie requirement and adequacy of patients with low mNUTRIC scores.

| Variable | Low Risk (mNUTRIC < 5) (n = 107) | | |
|---|---|---|---|
| | **Inadequate nutrition (n =6 3)** | **Adequate nutrition (n = 44)** | ***p* value** |
| Required calorie, kcal | 1575.44 ± 326.98 | 1372.10 ± 253.86 | 0.001 |
| Calorie adequacy, % | 40.38 ± 15.32 | 92.54 ± 17.70 | <0.001 |
| EN within 5 days, n(%) | 10 (15.9) | 10 (22.7) | 0.371 |
| PN supplement within 5 days, n(%) | 27 (42.9) | 42 (95.5) | <0.001 |

mNUTRIC: modified NUTrition Risk in Critically ill; EN: enteral nutrition, PN: parenteral nutrition.

**Table S4.** Clinical outcomes of patients with low mNUTRIC scores.

| Variables | Low Risk (mNUTRIC < 5) (n = 107) | | |
|---|---|---|---|
| | **Inadequate nutrition (n = 63)** | **Adequate nutrition (n = 44)** | ***p* value** |
| In-hospital mortality, n(%) | 5 (7.9) | 10 (22.7) | 0.030 |
| 30-day mortality, n(%) | 4 (6.3) | 8 (18.2) | 0.068 |
| MV-free day, median (Q1, Q3), d | 28 (19.0, 29.0) | 27 (19.3, 28.0) | 0.149 |
| ICU-free day, median (Q1, Q3), d | 25 (14.0, 27.0) | 25.5 (14.3, 27.0) | 0.771 |
| HLOS, median (Q1, Q3), d | 19 (14.0, 37.0) | 20.5 (14.3, 31.0) | 0.768 |
| IAI, n(%) | 12 (19.0) | 13 (29.5) | 0.207 |
| Pulmonary complication, n(%) | 34 (54.0) | 20 (45.5) | 0.386 |
| Wound complication, n(%) | 19 (30.2) | 13 (29.5) | 0.946 |
| Post-operative leak, n(%) | 6 (9.5) | 6 (13.6) | 0.507 |
| Infection, n(%) | 31 (49.2) | 27 (61.4) | 0.214 |

mNUTRIC: modified NUTrition Risk in Critically ill; MV: mechanical ventilation, ICU: intensive care unit; HLOS: hospital length of stay; IAI: intra-abdominal infection.
